# Preliminary Study of the Bactericide Properties of Biodegradable Polymers (PLA) with Metal Additives for 3D Printing Applications

**DOI:** 10.3390/bioengineering10030297

**Published:** 2023-02-27

**Authors:** Anyul López-Camacho, Dulce Magaña-García, María José Grande, Daniel Carazo-Álvarez, M. Dolores La Rubia

**Affiliations:** 1 Chemical, Environmental and Materials Engineering Department, University of Jaén, 23071 Jaén, Spain; 2 Smart Materials 3D, Polígono Industrial El Retamar, 7, 23680 Jaén, Spain; 3 Health Sciences Department, University of Jaén, 23071 Jaén, Spain; 4 Mechanical and Mining Engineering Department, University of Jaén, Campus Las Lagunillas s/n, 23071 Jaén, Spain

**Keywords:** anti-bacterial agents, polymer, PLA, 3D print, protective materials, *Listeria monocytogenes*, *E. coli*, industrial process

## Abstract

Plastic is a highly used material in various sectors. Due to its plentiful availability in the environment, microorganism surface contamination is a risk. The aim of this work is to achieve bactericidal capacity in plastics that reduces the microorganism’s colonization risk and, consequently, reduces the chances of having an infection with *E. coli* and *Listeria monocytogenes* bacteria. Using polylactic acid (PLA) as the polymeric matrix, mixtures in concentrations of metal additive of ions of silver (Ag) R148 and S254 in 1% and 2% have been studied and manufactured. The materials are developed on an industrial scale through a process that proceeds as follows: (I) a mixture of polymer and additive in a double-screw compounder to obtain the compound in different concentrations, (II) the manufacture of filaments with a single-screw extruder, (III) 3D printing parts. Therefore, materials are evaluated in the form of powder, pellets and printed pieces to ensure their antibacterial effectiveness throughout the manufacturing process. The results of the research show antibacterial effectiveness for *E. coli* and *Listeria monocytogenes* of metal additives and polymeric compounds for all manufacturing phases on an industrial scale, with the effectiveness for additive R148 predominating at a concentration of 2%, demonstrating its microbial efficacy on surfaces with potential application in medicine.

## 1. Introduction

Infectious diseases in humans, animals and plants are mainly caused by bacteria in comparison with fungi, parasites, protozoa and even viruses [1]. In recent decades, the World Health Organization (WHO), the European Centre for Disease Prevention and Control (ECDC) and the US Centers for Disease Control and Prevention (CDC) have alerted about the spread and resistance of bacteria as a public health problem [2]. The uncontrolled spread of microorganisms is related to transmission mechanisms, considering air as a transport medium in natural and artificial environments [3], which causes 80% of microbial diseases due to contaminated surfaces [4]. Consequently, it is essential to focus on the development of new materials with antimicrobial properties to fight bacterial spread. 

Materials science can offer an alternative for the elimination of infections on surfaces. Polymers are currently the most widely used materials in various applications, such as medicine, packaging and automotive applications. This wide availability makes it very interesting that they could be provided with antimicrobial capacity through the addition of additives and/or fillers. Several authors have studied the microbial capacity in polymers with the addition of metallic materials such as silver, copper, cobalt, zinc, zirconium [5,6,7,8], photocatalytic ${\mathrm{TiO}}_{2}$ [9] and other non-metallic materials such as acrylic monomers and polymeric biocide polyhexamethylene guanidine hydrochloride [10,11], quinine [12], graphene [13,14] and lignin [15]. Materials based on metal ions, or their nanoparticles, have been widely studied and used over the centuries for their antimicrobial properties. The mechanisms of action against bacteria of metals are attributed to the formation of reactive oxygen species (ROS), membrane disruption and interactions with DNA and proteins. Furthermore, the bactericidal behavior of metal-based materials differs from species and often from strain for each bacterium [16]. Previous research focused on the antimicrobial mechanisms of metal-based polymers such as silver (Ag) [5,17], copper (Cu) [18,19], gold (Au) [20,21] or zinc (Zn) [22]. These works were carried out on different polymeric matrices and bacterial strains, analyzing the influence of different parameters on bacterial capacity, e.g., particle size: V. Tomas et al. [17] evaluated the antibacterial effectiveness of hydrogels prepared with Ag nanoparticles tested on *E. coli*, verified that for smaller particle size, more contact with bacteria causing faster inhibition. V. Demchenko et al. [5] studied the effect of percentage concentrations of Ag nanoparticles in a polylactide matrix, reaching bacterial inhibition with 2%; higher concentrations are not necessary for bactericidal effectiveness for *E. coli* and *S. aureus.* Moreover, N. Gowriboy et al. [18] demonstrated larger zones of inhibition for higher percentages of Cu on *Pseudomonas aeruginosa*, *E. coli*, *Staphylococcus aureus* and *Enterococcus faecali,* and S. P. S. Chowdhury et al. [20] investigated the effect of Au nanoparticles in biopolymers, proving bactericidal efficacy with the lowest doses. Another issue is the ratio between the bactericidal capacity and the intervening strain. O. V. Hopta et al. [19] analyzed bacterial inhibition in low-density polyethylene composites with Cu nanoparticles and verified that bactericidal effectiveness depends on the bacterial family studied. On the other hand, the dispersion in the matrix and surface distribution of the material also influence behavior. According to T. Jayaramudu and associates [21], a strong inhibition against *S. pyogenes* and *E. coli* of hydrogel and Au composites was probed due to the homogeneous dispersion of Au in the matrix. Finally, A. C. Ciubotariu et al. [22] demonstrated higher inhibitions of bacteria for pure Zn for thermoset polymers. 

Among the heavy metals, silver has been ranked with the highest microbial effectiveness [23]. It has been extensively studied as an antimicrobial material in polymers under controlled laboratory conditions [5,7,24,25,26,27], proving its bactericidal effectiveness on different classes of bacteria: *S. aureus*, *E. coli* and *Streptococcus*. The mechanisms of action are based (i) on cell membrane damage and, as a result, on the alteration of cellular functions and (ii) on the formation of free radicals and cellular damage by oxidative stress ROS [28,29,30]. 

Concerning the application sector, 3D printing includes several technologies, such as stereolithography (SLA), Fused Deposition Modeling (FDM), selective laser sintering (SLS), direct metal laser sintering (DMLS) and poly-jet 3D printing [31]. This allows the application of a wide range of materials, such as metals [32], ceramics [33], polymers [34], biopolymers [35] and others. Three-dimensional printing is revolutionizing traditional production systems, enabling large-scale prototyping and machining of customized objects in local areas with minimal waste. The replicability of the industrial model is global; a three-dimensional digital file, a thermoplastic material and a 3D printer are required for the manufacture of objects. The representative advantages of 3D printing are the globalization of models through digital files, available anywhere in the world in seconds, allowing for printing, modifying and updating prototypes [36], as well as the reduction of global transportation, delivery deadlines, energy costs and, therefore, the environmental footprint. As regards the 3D-printing market, growth rates are exponential. In 2016 the global market size was approximately USD 5 billion; in 2019, it was approximately USD 20 billion. By 2025, it is estimated to be more than USD 35 billion [37]. For this reason, adopting a material with antimicrobial properties for 3D printing offers advantages due to its wide scope and many applications, specifically in medicine [37,38,39] and odontology due to biopolymers [40]. Several researchers have focused on achieving antimicrobial properties in 3D-printing materials using silver salt solution methods [41], silver nitrate solution [42], silver nanoparticles coatings [43] and evaporation-induced self-assembly (EISA) method of silver nanoparticles [44].

Several studies have focused on PLA/Ag composites [42,45,46,47,48]; however, the use of clay-coated Ag for the protection, dispersion and controlled release of silver ions for a molten blend with PLA and evaluation of the bactericidal efficacy throughout the manufacturing process in industrial settings (i) powder, (ii) compound and (iii) printed part remains unexplored. This study is necessary (1) for bacterial evaluation in an industrial-scale (non-laboratory) manufacturing conditions in three thermal steps: polymer blending by compounder melting, pellet-to-filament transformation in an extruder and filament-to-filament transformation into final parts through additive manufacturing technology, and (2) to evaluate bacterial effectiveness in an unconventional and scalable application such as 3D printing (FDM).

Considering that PLA polymeric compounds activated with silver ions coated with clay have not been tested for their microbial capacity in a whole production line and other studies [11,19,22,27,43,47] have demonstrated microbial activity in plastics, the present research proposes to study their effectiveness against *Listeria monocytogenes* and *E. coli* bacteria and to try to demonstrate that the proposed materials fit in the same microbial line.

## 2. Materials and Methods

### 2.1. Polymer, Additives and Bacteria

Amorphous grade polylactic acid (PLA) was obtained from Ingeo (Ingeo TM Bioplastic, EE. UU). Clay-coated silver ion microparticles of different thicknesses, R148 (R) and S254 (S), were obtained from Smart Materials 3D (Smart Materials 3D Printing, SL, Jaén, Spain). The final particle size distribution of the additives was determined using Malvern Mastersizer 2000 equipment. Obtaining the values of D10, D50 and D90 means that 10%, 50% and 90% of the sample are smaller than this size value, respectively.

The bacteria used in the study were *Listeria monocytogenes*, a common Gram-positive bacterium, and *E. coli*, a common Gram-negative bacterium; for each bacterium, PALCAM and EMB selective media were used, respectively. EMB differential selective medium for the isolation of coliforms in accordance with the standard ISO 21150 [49,50] and USP is prepared with 15 g/L EMB Agar of Sharlau, 10 g/L of lactose, 0.065 g/L of methylene blue, 10 g/L of gelatin peptone, 0.4 g/L of eosin and 2.0 g/L dipotassium phosphate; moreover, selective and differential solid medium PALCAM is prepared in accordance with the standard ISO 11290-1 [51] and 11290-2 [49] is prepared with 39 g/L PALCAM of Agar of Sharlau, 10 g/L of mannitol, 0.5 g/L of glucose, 0.8 g/L of esculin, 0.5 g/L of ferric ammonium citrate, 15 g/L of lithium chloride, 0.08 g/L of phenol red, 3.0 g/L of yeast extract and PALCAM selective supplement. Finally, TSB Agar medium was prepared of Sharlau of tryptone and soybeans according to the harmonized method of pharmacopeias and standards ISO 9308-1 [52], 22717 [53] and 22964 [54] and saline solution. All reagents were obtained from (Sharlab S.L, Sentmenat, Spain) and used as received.

### 2.2. Preparation of PLA and Silver Additives Compounds

**Figure 1:**
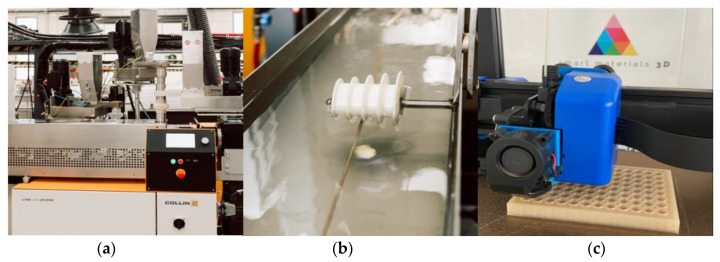
Industrial manufacturing process at Smart Materials 3D company (**a**) Compounder material mixing by melting; (**b**) Composite filament extrusion; (**c**) 3D printing of parts.

The polymer composites were manufactured under industrial conditions in the facilities of Smart Materials 3D company in three phases: compound, filament and 3D printing (Figure 1a–c). Initially, intrinsic moisture was removed from the materials in industrial dehumidifier for PLA at 60 °C for 8 h and for R148 and S254 additives at 100 °C for 4 h. Later, the mixtures were made by the melting method, the experimental program and mixtures of PLA/R and PLA/S as a percentage of 1 and 2%, respectively. Compounds PLA/R1, PLA/R2, PLA/S1 and PLA/S2 were obtained by blending in Compounder Collin (COLLIN Lab & Pilot Solution GmbH) (D = 25 mm, L/D = 48), twin screw co-rotating extruder for homogenization and dispersion of additives in the polymeric matrix, with a temperature ramp between 180 °C to 210 °C and an angular speed of 140 rpm. Then, the composite in pellet form was processed in a single-screw extruder for molding into filament form. Machine reached a temperature ramp between 180 °C to 210 °C and angular speed of 15 rpm. Finally, by additive manufacturing technology, Fused Deposition Modeling (FDM), the parts useful for characterization were printed. The 3D printing equipment used was Artillery GENIUS according to standard UNE-EN 116005 [55]; the printing parameters optimal for PLA, according to the manufacturer, are shown in Table 1:

### 2.3. Bacterial Growth Conditions

Bacterial growth was carried out according to [56,57]; each EMB and PALCAM medium, previously sterilized in autoclave at 121 °C for 20 min, is poured into Petri dishes. TSB medium is characterized by high nutrient content and is suitable for the growth and cultivation of a wide variety of demanding bacteria in liquid media. It is poured into 20 tubes at a rate of 5 mL/tube once it has been sterilized and autoclaved at 121 °C. The saline solution implemented in the bacteria count, as well as the strains *Listeria monocytogenes* and *E. coli*, are stored in cold storage until use in the laboratory. 

### 2.4. Microscopy

The samples examined by scanning electron microscopy were prepared from the microparticulate powder additives R and S, a compound in the form of pellets and rectangular printed parts, to evaluate the dispersion of the particles in the polymeric matrix for the initial and final steps of the industrial process. A MERLIN Carl Zeiss Scanning Electron Microscope (SEM) was used with a secondary electron (SE) image resolution of 0.8 nm at 15 Kv, 1.4 nm at 1 kv and 2.4 nm at 0.2 Kv.

### 2.5. Particle Size

The particle size of the additives was determined by laser diffraction technique using the Mastersizer 3000 equipment, which has an accuracy from 10 nm to 3.5 nm in particle distribution. It has a sequential combination of red and blue light, the latter being a solid state 10 mW advanced optics.

### 2.6. FTIR Spectroscopy

Infrared spectra were recorded by ATRFTIR (Vertex 70 Bruker equipment, Billerica, MA, USA) with a spectral resolution of 4 cm^−1^. FTIR spectra were collected from 400 cm^−1^ to 4000 cm^−1^. The samples analyzed for this assay were PLA as a reference of untreated material and PLA/R2, PLA/S1 and PLA/S2 composites, all in pellet form.

### 2.7. Evaluation of Bacterial Activity in Powder

For tests, following the methodology proposed by Caballero et al. [58] with particulate additives, positive controls (C) were prepared: one for *Listeria monocytogenes* and one for *E. coli* diluted in the following ratio of 1/100 with respect to overnight cultivation. For treated concentrations (T), mixtures were prepared 2 mL of BHI + 10 µL of each additive R and S (for 1% performance) + 20 µL of each corresponding strain. After that, the dilutions are sown (for example, C1:100 µL of the base, C2:100 µL of the C1, C3:100 µL of the C2, C4:100 µL of the C3) to verify the inhibition of bacterial growth by counting colony forming units (CFU) each test was performed in triplicate, grown on a plate at 0 h and 24 h.

### 2.8. Evaluation of Bacterial Activity in Compound

For the evaluation of compounds in pellet form, 5 units of each compound are individually deposited into tubes containing 5 mL of TSB + 100 µL of overnight strains of *Listeria monocytogenes* and *E. coli*. Bacterial evaluation is performed in two formats: (i) Dilutions are seeded in Petri dishes and incubated at 37 °C for bacterial count at 24 h. (ii) The dilutions are placed in a microtiter plate to measure the effectiveness of the bactericide by analyzing turbidity through absorbance measurement in a Spectro-photometer using a wavelength of 490 nm. Moreover, tests are performed on the positive controls of *Listeria monocytogenes* and *E. coli* with incubation at 37 °C; the method for microbiological analysis for triplicate was carried out according to Ortega et al. and Martínez et al. [59,60] by performing serial dilutions from 10^0^ to 10^2^ and subsequently seeding plate for viable cell counts at 0 h and 24 h. 

### 2.9. Evaluation of Bacterial Activity in 3D-Printed Parts

**Figure 2:**
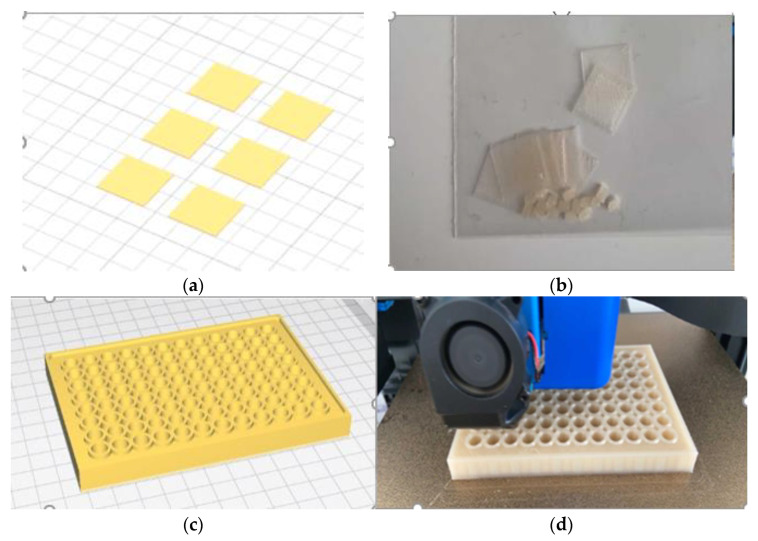
Sheets and microtiter plate; 3D printed. (**a**) STL file of sheets (**b**) Printed sheets (**c**) Microtiter plate STL file (**d**) Printed microtiter plate.

For the evaluation of the bactericidal activity of each compound, the method for microbiological analysis was obtained according to Ortega et al. and Martínez et al. [59,60] on printed parts carried out in two forms: (i) printed pieces in the form of square sheets, and (ii) of printed microtiter plate. (i) Three square sheets for material of dimensions 1.0 × 1.0 × 0.2 mm (Figure 2a,b) were evaluated through the measurement in mm of the inhibition halo formed around the printed piece. For this test, the films were deposited on plates PALCAM and EMB, previously inoculated with *Listeria monocytogenes* and *E. coli,* respectively, by means of exhaustion using a sterile swab. Subsequently, composite sheets R (PLA/R1, PLA/R2) were cast on top of the plate, and composite sheets S (PLA/S1 and PLA/S2) were deposited at the bottom; in the center of the plate, a control film C (PLA) was placed, which contains no additive. The plates were 24 h incubated in an oven at 37 °C, obtaining the results by the analysis of the inhibition halo.

(ii) In the form of a printed microtiter plate (Figure 2c,d), the bactericidal evaluation was carried out according to the turbidity and, therefore, the absorbance was recorded in the wells, with reading by means of a spectrophotometer at a wavelength of 490 nm. For printed plates, three plates per material were printed (identical in shape, structure and similarity to the micro titration plates used in the laboratory) with compounds PLA/R1, PLA/R2, PLA/S1, PLA/S2, each of the strains was diluted in saline solution at a ratio of 1/100 (A). Plates were inoculated with 200 μL of the strain in each well using decreasing concentrations (1/10, 1/100, 1/1000 and 1/10,000) in saline solution. One of the wells was inoculated as a positive control, only with the strain without additive, and another well was used as a negative control, only with TSB culture medium.

## 3. Results and Discussion

### 3.1. Microscopy

**Figure 3:**
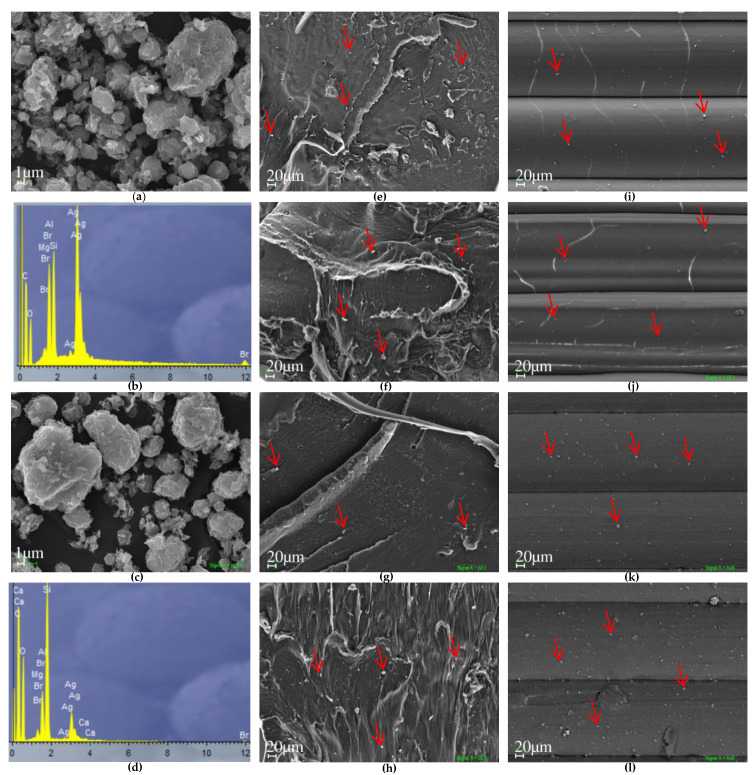
Scanning Electron Microscopy, SEM (**a**) Morphology additive R148 (**b**) EDX R148 (**c**) Morphology additive S254 (**d**) EDX S254 (**e**) Particle distribution PLA/R1 in pellets (**f**) Particle distribution PLA/R2 in pellets (**g**) Particle distribution PLA/S1 in pellets (**h**) Particle distribution PLA/S2 in pellets (**i**) Particle distribution PLA/R1 in 3D-printed part (**j**) Particle distribution PLA/R2 in 3D-printed part (**k**) Particle distribution PLA/S1 in 3D-printed part (**l**) Particle distribution PLA/S2 in 3D-printed part. Note: The red arrows indicate the particles of additive.

In the images (Figure 3a,c) of the particulate additives, it is observed that R and S have a predominantly spherical morphology; likewise, a size distribution is observed between 1.6–13.1 µm for additive R and 1.5–12.5 µm for additive S, the predominant chemical elements in the EDX analysis are Ag, Si, Mg, Al and Br for both (Figure 3b,d). In the evaluation of the silver ions distribution in the PLA matrix (Figure 3e–l), it is observed that for all compounds, there is a significant distribution of the additives over the entire surface area of the pellet and the filament, this allows the potential bactericidal effect to act homogeneously on end-pieces. The industrial melt blending process allows the homogenization of the particles; in addition, transformation processes such as extrusion and 3D printing maintain the homogenization without affecting the final active properties of the material.

### 3.2. Particle Size

**Figure 4:**
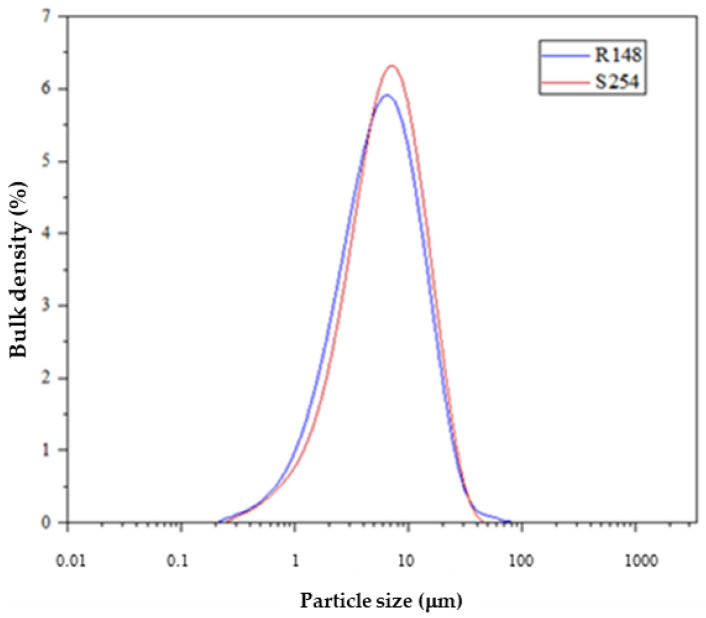
Particle size distribution for R148 and S254 additives.

According to several authors [17,61], the behavior of silver depends on the particle size, with the nanoscale being the most effective size. In the case of R and S additives, the silver ions are in nano size; however, they are coated with ceramic material so as to avoid exposure to the silver. It can be observed in Figure 4 that R and S have a D90 of 16.5 µm and 17.3 µm, respectively, keeping a similar particle size and distribution. The coating thickness turns out to be the main factor; the additive S has less coating, and therefore, the silver is more exposed.

### 3.3. FTIR Spectroscopy

**Figure 5:**
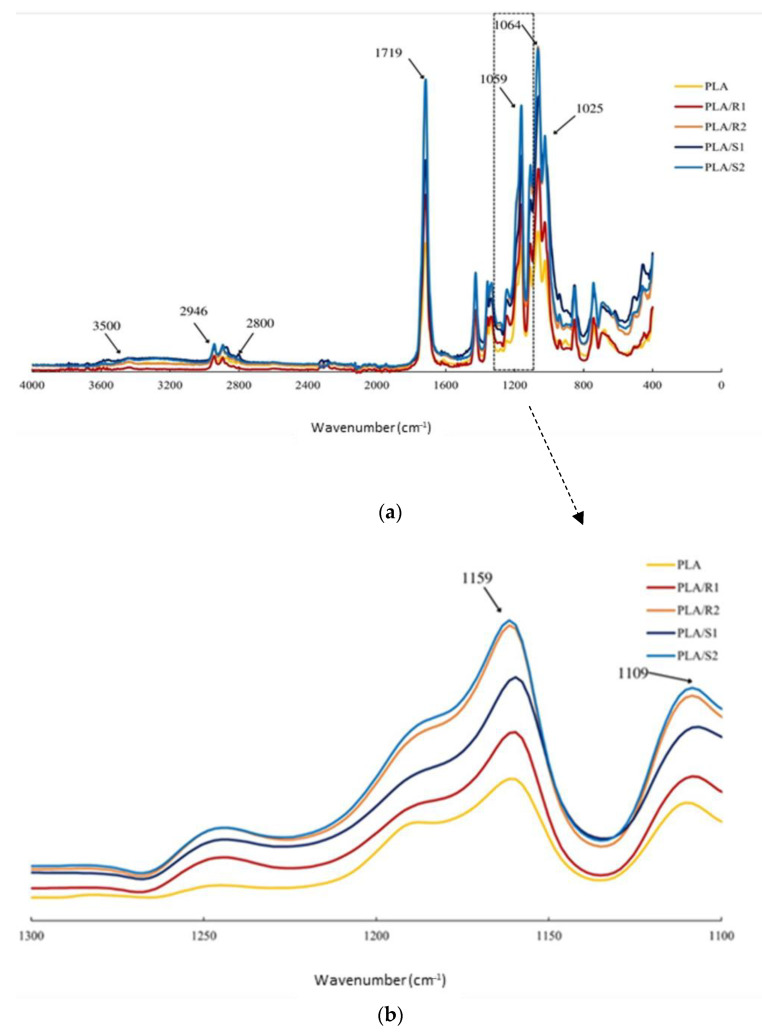
FTIR Spectrum of PLA, PLA/R1, PLA/R2, PLA/S1 and PLA/S2 (**a**) 400–4000 cm^−1^ region and (**b**) 2800–300 cm^−1^ region.

In the FTIR spectrum (Figure 5), for the PLA sample, the typical bands 2800–3000 $cm^{-1}$ appear, corresponding to structure with C-H bond of asymmetric and symmetric stretching vibrations. [62]. On the other hand, the most predominant peaks of 1719 $cm^{-1}$, 1100–1300 $cm^{-1}$ is related to the vibration of the C=O and C-O-C peaks of PLA; furthermore, a weak band appears above 3400–3500 $cm^{-1}$ corresponding to the stretching vibration of the terminal hydroxyl group (-OH) of PLA and the residual H_2_O molecules in the system [63]. Moreover, we can observe in Figure 5 that the strong absorption peaks of the compounds (PLA/R1, PLA/R2, PLA/S1 and PLA/S2) with respect to pure PLA show no obvious differences. A similar phenomenon has been studied by Li et al. [64], in which the addition of nano-Ag did not alter the molecular structure and interaction; however, the intensity of the band was increased (Figure 5b). This phenomenon can be explained by the fact that the nucleation rate and density were higher than those of PLA.

### 3.4. Bacterial Activity in Powder

First, bacterial growth was verified in Petri dishes *Listeria monocytogenes*: $C_{1}$ + L, $C_{2}$ + L, $C_{3}$ + L, $C_{4}$ + L and *E. coli*: $C_{1}$ + E, $C_{2}$ + E, $C_{3}$ + E, $C_{4}$ + E to 0 and 24 h. A decrease in the count of microorganisms corresponding to each dilution was demonstrated, which proves the effectiveness of the compound. In the colony count, it is calculated with the method of CFU; the bacterial growth values at 24 h for *Listeria monocytogenes* is 5.60 × $10^{8}$ CFU/mL and of 3.00 × $10^{8}$ for *E. coli*.

**Figure 6:**
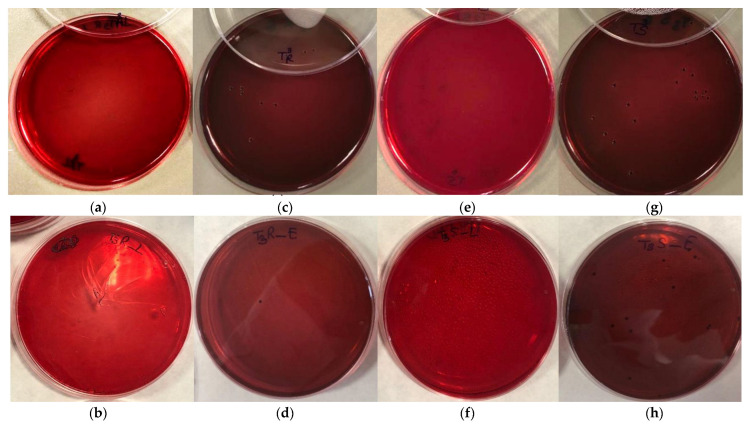
Antibacterial activity of (**a**) Additive R with *Listeria monocytogenes* at 0 h (**b**) Additive R with *Listeria monocytogenes* at 24 h (**c**) Additive R with *E. coli* at 0 h (**d**) Additive R with *E. coli* at 24 h (**e**) Additive S with *Listeria monocytogenes* at 0 h (**f**) Additive S with *Listeria monocytogenes* at 24 h (**g**) Additive S with *E. coli* at 0 h (**h**) Additive S with *E. coli* at 24 h.

The results of the study with the R, inhibition of bacterial growth is evidenced in plates with *Listeria monocytogenes*: $R_{1}$ + L, $R_{2}$ + L, $R_{3}$ + L, $R_{4}$ + L to 0 h (Figure 6a), that means that, from time zero, effectiveness is shown, thus corroborating the results with their respective replication. After seeding and incubating the plates for 24 h to 37 °C, verification is performed, showing that the additive does not allow bacterial growth (Figure 6b) in any dissolution. In the colony count, by calculating the number of CFU/mL, bacterial growth values at 24 h for *Listeria monocytogenes* are 0 CFU/mL. On the other hand, for *E. coli*, bacterial growth is evident from time 0 (Figure 6c) on plates $R_{1}$ + E, $R_{2}$ + E, $R_{3}$ + E, although in smaller quantities than for control plates at equal concentrations. However, after 24 h (Figure 6d), the additive has an antibacterial effect, eliminating the viable cells of *E. coli* and reducing the values of CFU/mL to undetectable limits.

Subsequently, the bactericidal effect of the S-type additive was evaluated for 0 h (Figure 6e); in this case, growth was observed *Listeria monocytogenes* for $S_{1}+L$ and $S_{2}+L$, however, for dilutions $S_{3}+L$ and $S_{4}+L$ no growth is visible. In contrast, in the 24 h results (Figure 6f), complete colony elimination is observed in all the plates: $S_{1}$ + L, $S_{2}$ + L, $S_{3}$ + L, $S_{4}$ + L with counts of CFU/mL equals zero. Finally, by verifying the behavior of additive S at *E. coli*, for 0 h (Figure 6g), bacterial growth is observed in a higher proportion than in the additive R. Nevertheless, in the 24 h results (Figure 6h), as opposed to the additive R, the S cannot completely eliminate the number of viable, but if it manages to significantly decrease its concentration from 2.39 × $10^{7}$ in control up to 2.30 × $10^{5}$ CFU/mL. All values are represented in Table 2:

The results demonstrate that R and S powder additives have high antimicrobial activity against *Listeria monocytogenes* and *E. coli* strains after incubation at 37 °C for 24 h. A characteristic of the R and S additives that favors bactericidal behavior is the particle size (as indicated in Section 3.2); according to V. Tomas et al. [17], particles smaller than 100 µm are more effective against bacteria. The reason, according to Oberdorster et al. [61], is that silver, in the form of ions or nanoparticles, can invade pathogens and induce binding to enzyme thiol groups or proteins, leading to metal disorder and the death of bacteria.

### 3.5. Bacterial Activity in Compound

**Figure 7:**
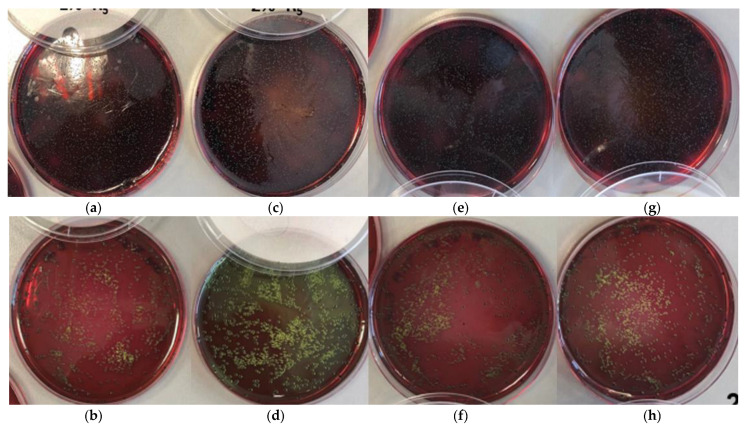
Antibacterial activity of (**a**) PLA/R1 compound with *Listeria monocytogenes* (**b**) PLA/R1 compound with *E. coli* (**c**) PLA/R2 compound with *Listeria monocytogenes* (**d**) PLA/R2 compound with *E. coli* (**e**) PLA/S1 compound with *Listeria monocytogenes* (**f**) PLA/S1 compound with *E. coli* (**g**) PLA/S2 compound with *Listeria monocytogenes* (**h**) PLA/S2 compound with *E. coli*.

For the verification of the activity in Petri dishes, first, the positive controls are visualized, ensuring that the colonies of each strain grow normally in their respective culture medium, and a decrease in colonies is also evident according to the serial dilution tested. The evaluation of bactericidal effectiveness in pellet format shows that in this format, the material does not have bactericidal activity for PLA/R1, PLA/R2, PLA/S1 and PLA/S2 compounds against *Listeria monocytogenes* and *E. coli*, as the number of viable colonies on the plate exceeds 300 colonies, as shown in Figure 7a–h, where each white dot on the Petri dish represents a colony of *Listeria monocytogenes* (Figure 7a,c,g,e) and each green dot represents a colony of *E. coli* (Figure 7b,d,f,h).

**Figure 8:**
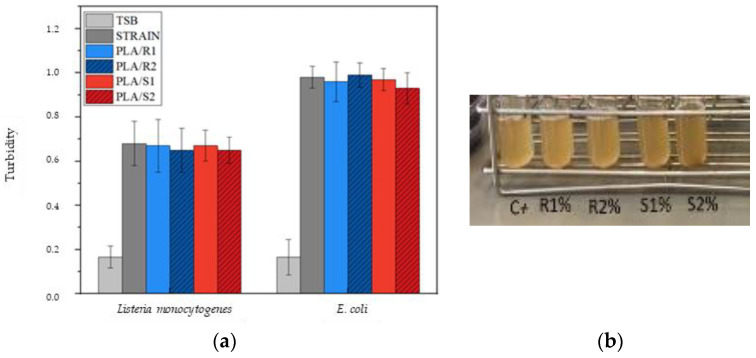
Bacterial activity of (**a**) compounds in compound form PLA/R1, PLA/R2, PLA/S1 and PLA/S2 microtiter plate assays for bacteria *Listeria monocytogenes* and *E. coli* (**b**) Tube turbidity. Standard deviation calculated with three replications per test.

At the same time, in the microtiter plate assays, the results obtained (Figure 8a) are consistent with the previous ones; in pellet form, the compounds PLA/R1, PLA/R2, PLA/S1 and PLA/S2 have turbidity (Figure 8b) resulting in a very mild inhibition of bacteria *Listeria monocytogenes* and *E. coli*.

### 3.6. Bacterial Activity in 3D Printing

**Figure 9:**
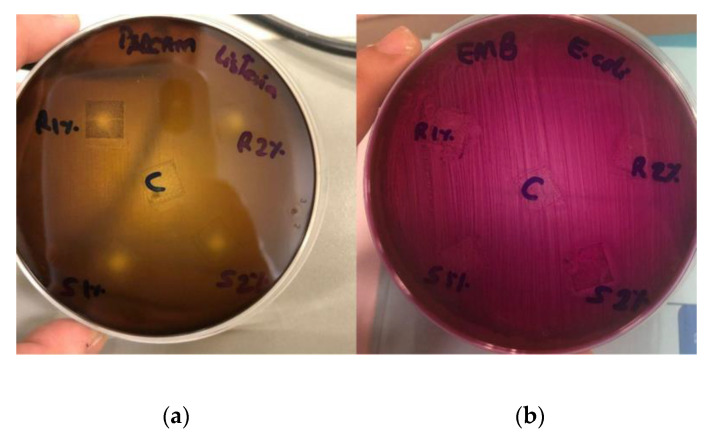
Antibacterial activity of compounds PLA/R1, PLA/R2, PLA/S1 and PLA/S2 and control on bacteria (**a**) *Listeria monocytogenes* and (**b**) *E. coli*.

The effectiveness of the sheets was evaluated by observing the inhibition halo. Figure 9a,b shows the experimental result of the films printed with the compounds of PLA/R1, PLA/R2, PLA/S1 and PLA/S2, and the control, where the inhibition of bacterial growth is evidenced at 24 h. Likewise, different geometric behavior of the inhibition halo was observed for each intervening strain, for *Listeria monocytogenes* is manifested in the center of the film in the form of a circle showing that the activity of the additive R in percent of 1% is less than the activity of the additive R as a percentage of the 2%. However, it can be evaluated that the additive S in percentages of 1 and 2% have little resistance. In addition, for *E. coli*, the inhibition halo is square-shaped, bordering the film, and as in the previous case, there is evidence of greater effectiveness of R additives with respect to S additives. The measurement of the inhibition halos in each of the samples is as follows: for *Listeria monocytogenes*, 6 mm (PLA/R1), 9 mm (PLA/R2), 5 mm (PLA/S1) and 8 mm (PLA/S2moreover, for *E. coli*, are: 12 mm (PLA/R1), 15 mm (PLA/R2), 10 mm (PLA/S1) and 14 mm (PLA/S2). These measures are presented in Table 3:

As several authors have previously stated [5,17], it is evident that a further increase in concentrations improves the antimicrobial activity of the R and S compounds. The results are consistent with another study [65] where it is evident that in gram-negative bacteria such as *E. coli*, inhibition is greater than in large-positive bacteria such as *Listeria monocytogenes*; this phenomenon is related to the cell wall structures and their composition since large negative bacteria have a lipopolysaccharide layer (1–3 µm of thickness) and peptidoglycans (~8 nm of thickness), which could facilitate the penetration of metal ions. On the other hand, large positive bacteria are coated with a much thicker peptidoglycan layer (~80 nm), which hinders the ingress of metal ions [28,65].

Another action mechanism of silver is related to the interaction with bacterial DNA, which leads to the destruction of the structure. DNA replication is disrupted by degeneration, which induces the interruption of bacterial reproduction [66]. These damages were analyzed by [25] H. Y. Chu et al. by means of a transmission scanning electron microscope (TEM), who observed the cell structure with the interaction of a silver-based antimicrobial material; they observed that the interaction resulted in the loss of cell cohesion and the outer membranes were severely destroyed, causing the outgrowth of cytoplasm and finally the death of vegetative cells of the cells *E. coli*. Evidently, the research results demonstrate that the bacteria-compound interactions PLA/R1, PLA/R2, PLA/S1 and PLA/S2 are influenced by Ag+ ion releases and their antibacterial activity.

In this respect, the materials studied demonstrate their bacterial efficacy with *E. coli* and *Listeria monocytogenes* bacteria belonging to the biofilm-forming microbiota, according to Rohatgi et al. [67] biofilm development includes four main stages: (i) reversible adhesion; (ii) irreversible adhesion and early structural development; (iii) maturation of the biofilm; and (iv) dispersal of the cells; this mechanism promotes surface adhesion, fostering infections. Some studies [68,69] have observed the presence of microbes in the form of biofilms in plastics, which is why the study of PLA/R1, PLA/R2, PLA/S1 and PLA/S2 compounds for surface disinfection is of functionality as a final product, which promotes the reduction of infections on surfaces.

**Figure 10:**
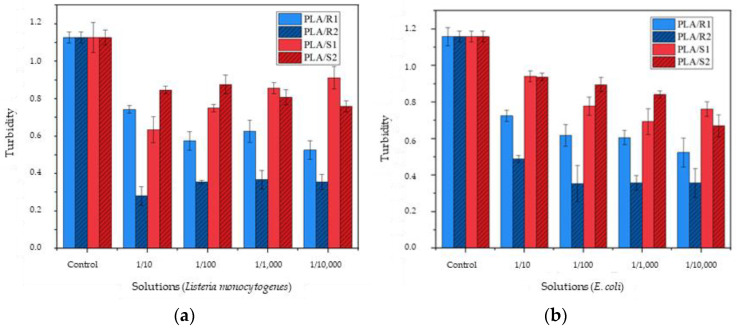
Bacterial efficacy of 3D-printed microtiter pieces for each composite PLA/R1, PLA/R2, PLA/S1 and PLA/S2 (**a**) *Listeria monocytogenes* (**b**) *E. coli.* Standard deviation calculated with three replications per test.

Finally, in the evaluation in microtiter plates printed by additive manufacturing of each compound PLA/R1, PLA/R2, PLA/S1 and PLA/S2, it is observed in Figure 10a,b that R additives are the most effective, having higher inhibition at 2% compared to compounds with S additive for the two strains of bacteria.

## 4. Conclusions

In the present study, the antibacterial capacity of additive polymers for use in 3D printing (FDM) is evaluated. This capacity of the proposed mixtures is analyzed both for the compound in pellet format and for the printed parts. The R and S additives have adequate particle sizes for this task. SEM images are used to observe the dispersion of the additives in the polymeric matrices, which is quite homogeneous.

Regarding the bacterial evaluation in powder format, the greatest effect is presented with R in a concentration of 2%, completely inhibiting the bacteria: *Listeria monocytogenes* and *E. coli*. For the compound format, a slight inhibition of bacterial growth is verified. Finally, in the printed test, plate assay and inhibition halos are observed for R and S in their respective percentages, presenting rectangular shapes in *E. coli* and circular shapes in *Listeria monocytogenes*. Similarly, bactericidal effectiveness is demonstrated in microtiter plates at concentrations of PLA/R1, PLA/R2, PLA/S1 and PLA/S2, keeping R as the predominant additive.

In conclusion, the results of the research show antibacterial effectiveness for *Listeria monocytogenes* and *E. coli* bacteria of metal additives and polymeric compounds for all manufacturing phases on an industrial scale, with the effectiveness for R predominating at a concentration of 2%.

## Figures and Tables

**Table 1:** 3D-Printing process settings for Artillery GENIUS printer.

Parameter	Value
Layer height (mm)	0.2
Nozzle diameter (mm)	0.4
Wall thickness (mm)	0.8
Fill (%)	100
Top and bottom layer number (Nº)	4
Printing temperature (°C)	215
Bed temperature (°C)	60

**Table 2:** Colony forming units (CFU) for Petri dishes: control, additive R148 and additive S254.

Sample	Control		Additive R		Additive S	
Bacteria/ Time (h)	*Listeria monocytogenes* (CFU/mL)	*E. coli* (CFU/mL)	*Listeria monocytogenes* (CFU/mL)	*E. coli* (CFU/mL)	*Listeria monocytogenes* (CFU/mL)	*E. coli* (CFU/mL)
0	$2.39\;\times \;10^{7}$	$2.90\;\times \;10^{6}$	0	$9.00\;\times \;10^{5}$	$1.80\;\times \;10^{6}$	$1.90\;\times \;10^{6}$
24	$5.60\;\times \;10^{8}$	$3.00\;\times \;10^{8}$	0	0	0	$2.30\;\times \;10^{5}$

**Table 3:** Bacterial inhibition halos of compounds PLA/R1, PLA/R2, PLA/S1 and PLA/S2 and control on bacteria *Listeria monocytogenes* and *E. coli*.

Bacteria/ Material	*Listeria monocytogenes* (mm)	*E. coli* (mm)
PLA/R1	6.0	12.0
PLA/R2	9.0	15.0
PLA/S1	5.0	10.0
PLA/S2	8.0	14.0

## Data Availability

Data are contained within the article.
